# A comprehensive overview of the effects of probiotics, prebiotics and synbiotics on the gut-brain axis

**DOI:** 10.3389/fmicb.2025.1651965

**Published:** 2025-10-17

**Authors:** Gizem Kezer, Spiros Paramithiotis, Khaoula Khwaldia, Iskandar Azmy Harahap, Martina Čagalj, Vida Šimat, Slim Smaoui, Walid Elfalleh, Fatih Ozogul, Tuba Esatbeyoglu

**Affiliations:** ^1^Department of Molecular Food Chemistry and Food Development, Institute of Food and One Health, Gottfried Wilhelm Leibniz University, Hannover, Germany; ^2^Department of Agricultural Biotechnology, Faculty of Agriculture, Kırşehir Ahi Evran University, Kırşehir, Türkiye; ^3^Laboratory of Microbiology, Department of Biological Applications and Technology, University of Ioannina, Ioannina, Greece; ^4^Laboratoire des Substances Naturelles (LSN), Institut National de Recherche et d’Analyse Physico-chimique (INRAP), BiotechPole Sidi Thabet 2020, Sidi Thabet, Tunisia; ^5^Research Organization for Health, National Research and Innovation Agency, Bogor, Indonesia; ^6^University Department of Marine Studies, University of Split, Split, Croatia; ^7^Laboratory of Microbial and Enzymatic Biotechnologies and Biomolecules, Center of Biotechnology of Sfax (CBS), University of Sfax, Sfax, Tunisia; ^8^Department of Biology, College of Science, Imam Mohammad Ibn Saud Islamic University (IMSIU), Riyadh, Saudi Arabia; ^9^Department of Seafood Processing Technology, Faculty of Fisheries, Cukurova University, Adana, Türkiye; ^10^Biotechnology Research and Application Center, Cukurova University, Adana, Türkiye

**Keywords:** probiotic, prebiotic, synbiotic, gut-brain axis, health

## Abstract

The gut-brain axis (GBA) represents a complex bidirectional communication system connecting the gastrointestinal tract and the central nervous system through neural, endocrine, immune, and metabolic pathways. Emerging evidence suggests that dietary interventions, particularly probiotics, prebiotics, and synbiotics, can influence the composition and activity of the gut microbiota, thereby modulating GBA function. Such modulation has been linked to potential benefits for cognitive performance, emotional regulation, and resilience against neurodegenerative and neuropsychiatric disorders. In addition, these interventions may contribute to immune homeostasis and the management of chronic conditions such as inflammatory bowel disease, irritable bowel syndrome, and multiple sclerosis. Nevertheless, the mechanisms underlying these effects and their long-term clinical relevance remain incompletely understood. In this narrative review, we systematically synthesize current clinical and preclinical evidence on the role of probiotics, prebiotics, and synbiotics in regulating the GBA. Particular attention is given to their impact on neurocognitive outcomes and systemic health, highlighting both the therapeutic potential and the existing gaps that warrant further investigation.

## Graphical abstract

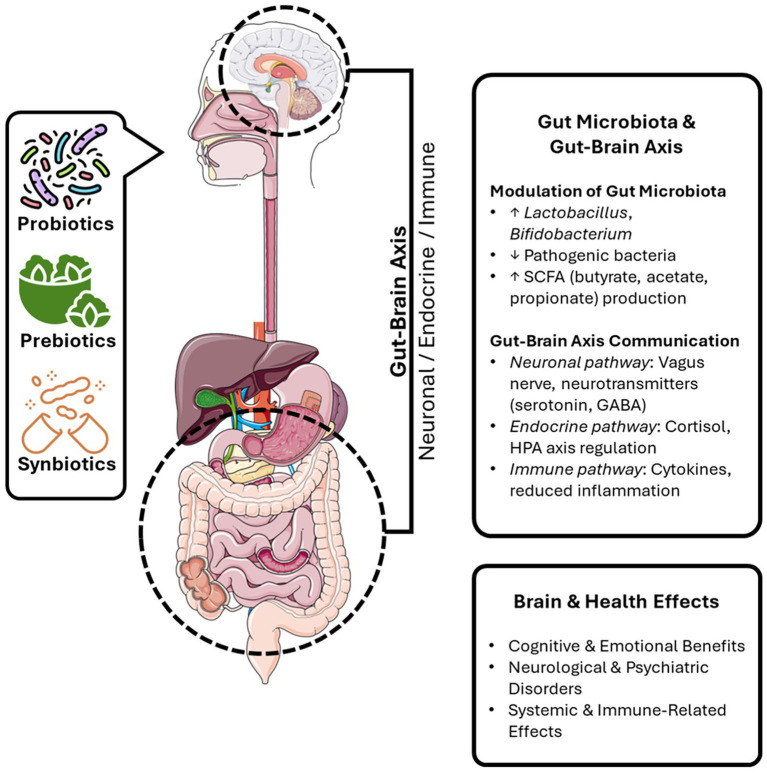

## 1 Introduction

The microbiota is the community of microorganisms colonizing the human body and interacting with the host. The mammalian gut alone contains around 2000 bacterial species, forming a complex ecosystem known as the gut microbiota (Góralczyk-Bińkowska et al., 2022; Maiuolo et al., 2021; Loh et al., 2024). This ecosystem is not randomly assembled; rather, it is shaped by selective pressures that reflect host genetics, diet, environment, and evolutionary history. In healthy adults, the gut microbiota is dominated by five phyla: Firmicutes (79.4%), Bacteroidetes (16.9%), Actinobacteria (2.5%), Proteobacteria (1%), and Verrucomicrobia (0.1%; Szymczak-Pajor et al., 2025; Maiuolo et al., 2021).

Members of the gut microbiota contribute to a wide range of metabolic functions by expressing enzymes and genes that facilitate nutrient conversion, energy harvest, and biosynthesis of essential compounds. These include amino acids, vitamins, short-chain fatty acids (SCFAs), and lipids, which play key roles in host physiology. Furthermore, the microbiota produces antimicrobial substances that protect against pathogenic colonization and supports intestinal barrier maturation and immune system regulation. A balanced and diverse microbiota is therefore critical for maintaining host health. Conversely, microbial diversity and community structure vary markedly among individuals due to factors such as mode of birth, early-life nutrition, lifestyle, pharmacological exposure, and genetic background (Morais et al., 2021; Maiuolo et al., 2021).

Recent insights suggest that the bioactive landscape of microbiota-derived metabolites is much more complex than previously assumed, with specialized peptides exhibiting antibacterial, immunomodulatory, and signaling roles (Shah and Shim, 2025; Shah et al., 2025). These discoveries highlight the microbiome not only as a determinant of host well-being but also as a source of novel therapeutic strategies.

The gastrointestinal tract remains the primary habitat for this microbial community, containing trillions of microorganisms, which outnumber host cells by nearly tenfold. Dysbiosis, defined as an imbalance of the gut microbiota, has been implicated in a range of metabolic, immunological, and neurological conditions (Verma et al., 2025; Bhagwat et al., 2025). The microbiota is now recognized as an active participant in host physiology, influencing systemic metabolism, immune development, and organ function.

One of the most studied aspects of host–microbe interaction is the bidirectional communication between the gut microbiota and the central nervous system (CNS), termed the microbiota-gut-brain axis. Although the gut and brain are anatomically distinct, multiple biological pathways facilitate this crosstalk, including neural (vagus nerve, enteric nervous system), immune, and endocrine signaling. Through the production of neurotransmitters, metabolites, and hormones, gut microbes are capable of modulating CNS activity (Liang et al., 2018; Loh et al., 2024; Ashique et al., 2024).

The microbiota-gut-brain axis thus represents a complex communication network that integrates microbial, immune, endocrine, and neural signaling to maintain homeostasis. However, perturbations in this system may contribute to the pathogenesis of neuropsychiatric and neurodegenerative diseases (Morais et al., 2021). Against this background, probiotics, prebiotics, and synbiotics have gained attention for their potential to beneficially modulate gut microbiota composition and function (Ansari et al., 2023). Probiotics are defined as live microorganisms that confer health benefits when consumed in adequate amounts, with *Lactobacillus* and *Bifidobacterium* among the most studied genera (Fekete et al., 2024; Ansari et al., 2023). Prebiotics, including galacto-oligosaccharides (GOS), fructo-oligosaccharides (FOS), and xylo-oligosaccharides, are nondigestible substrates that selectively stimulate the growth or activity of beneficial microbes (Ansari et al., 2023; Fekete et al., 2024). Synbiotics combine probiotics and prebiotics to act synergistically, with benefits ranging from improved digestion to potential roles in neuropsychiatric health (Markowiak and Śliżewska, 2017; Fekete et al., 2024; Ansari et al., 2023).

Although preclinical and clinical evidence suggests that microbiota-targeted interventions can influence CNS outcomes such as mood, cognition, and stress resilience (Chudzik et al., 2021; Radford-Smith and Anthony, 2023; Fekete et al., 2024), findings remain inconsistent. Randomized controlled trials have reported both positive effects and null results, reflecting strain-specificity, dosage variability, treatment duration, and methodological differences (Hofmeister et al., 2021; Alli et al., 2022; Nikolova et al., 2019). Moreover, the strong effects observed in animal studies have not always translated to humans (Forssten et al., 2022; Slykerman et al., 2025). Limitations including small sample sizes, heterogeneous populations, and lack of standardized protocols reduce the generalizability of current findings. There is also debate about whether beneficial effects arise from direct microbial activity, modulation of host–microbe interactions, or downstream immunological and metabolic changes.

Taken together, these controversies underscore that while probiotics, prebiotics, and synbiotics are promising as adjunctive approaches to CNS disorders, their clinical efficacy remains inconclusive. Alongside established strategies such as diet modification and fecal microbiota transplantation, these interventions represent an emerging frontier in brain-gut research. Future investigations should focus on large-scale, multicenter clinical trials, and mechanistic studies to clarify pathways of action and define clinical relevance. This review therefore aims to provide a critical synthesis of the current literature, highlighting mechanisms, therapeutic potential, limitations, and research gaps concerning the effects of probiotics, prebiotics, and synbiotics on the gut-brain axis. In preparing this narrative review, we performed a comprehensive search of relevant peer-reviewed literature using databases such as PubMed, Scopus, and Google Scholar. Articles were identified through combinations of keywords including probiotics, prebiotics, synbiotics, gut-brain axis, and neurocognitive health. Priority was given to recent publications (within the past 10–15 years), landmark studies, and mechanistic reports that provide insight into underlying pathways. Reference lists of pertinent papers were also examined to ensure inclusion of additional relevant works. Figures were generated and adapted using Microsoft PowerPoint and BioRender, based on data synthesized from the reviewed studies. This approach ensures both breadth and depth of coverage, while maintaining the narrative character of the review.

## 2 Probiotics

Among the many microbes that are an essential part of human life, probiotics have been recognized and extensively studied for their health benefits, particularly for the prevention of various gastrointestinal, metabolic and chronic diseases (Swanson et al., 2020). To be classified as a probiotic, the strain must be non-pathogenic, non-toxic, free from transferable antibiotic resistance genes, adequately characterized, tested for safety and technical characteristics for the intended use, maintain a viable population throughout its shelf life, and be proven to confer health benefits (Hill et al., 2014). In addition, a suitable and efficient probiotic must fulfill several functional criteria, such as maintenance of genetic integrity, resistance to exposure to low pH and bile salts, effective adherence to intestinal epithelial cells, production of beneficial metabolites, stability under industrial processing conditions, and the ability to multiply efficiently in the intestinal environment (de Melo Pereira et al., 2018).

Antimicrobial resistance must be considered in the safety assessment of probiotics, as strains carrying transmissible antibiotic resistance genes (ARG) could trigger horizontal gene transfer (HGT) in the gut, meaning that ARG can be transferred to pathogenic bacteria in the gut microbiome, resulting in drug-resistant strains. Therefore international FAO/WHO guidelines and the EFSA Qualified Presumption of Safety (QPS), emphasize that probiotics intended for human use must be free of ARG, while intrinsic, non-transferable resistance is generally acceptable. For example, many lactobacilli are intrinsically resistant to vancomycin due to their cell wall characteristics that result in reduced vancomycin binding. The current best practice recognizes the importance of rigorous screening through a multi-step approach: Whole genome sequencing for species identification and in silico ARG/mobilome analysis; phenotypic susceptibility testing to confirm resistance patterns; and, if indicated, conjugation or transmissibility testing to assess HGT potential [Tóth et al., 2021; Merenstein et al., 2023; Byakika et al., 2019; EFSA Panel on Contaminants in the Food Chain (CONTAM), 2018; EFSA BIOHAZ Panel et al., 2025; FAO/WHO, 2006].

Throughout history, fermented foods such as yoghurt and fermented vegetables have been the main source of probiotics in the human diet. Today, probiotics are available as dietary supplements and are used to fortify foods in a variety of strains and dosages. The psychobiotic effects of probiotics are strain- and dose-specific, and have been studied to identify potential therapeutic applications and to develop more efficient delivery systems. The most commonly used probiotics belong primarily to the genera *Lactobacillus* and *Bifidobacterium*. Common species include *Lacticaseibacillus casei* (*L. casei*), *Lactiplantibacillus plantarum* (*L. plantarum*), *Lactobacillus acidophilus* (*L. acidophilus*), *Lactobacillus helveticus* (*L. helveticus*), *Lacticaseibacillus rhamnosus* (*L. rhamnosus*), *Bifidobacterium longum* (*B. longum*), *Bifidobacterium bifidum* (*B. bifidum*), and *Bifidobacterium breve* (*B. breve*). Other probiotic strains that are gaining interest are *Saccharomyces cerevisiae* var. *boulardii* (a beneficial yeast), *Streptococcus thermophilus*, and strains of *Bacillus*, *Lactococcus*, *Enterococcus* and some *Escherichia coli* (*E. coli*) (Sarita et al., 2025).

### 2.1 Mechanisms

Probiotics (sometimes referred to as *psychobiotics* in this context) can modulate communication between the microbiota, gut and brain through multiple pathways. They influence neuronal signaling (e.g., via the vagus nerve and the enteric nervous system), hormonal responses (such as modulation of cortisol and the HPA axis) and immune activity (including cytokine regulation and inflammation; Mörkl et al., 2020). Certain probiotic bacteria, particularly *Lactobacillus* and *Bifidobacterium* species, produce neuroactive compounds - for instance, neurotransmitters such as GABA and serotonin, as well as short-chain fatty acids - that can affect brain function (Rahmannia et al., 2024). In addition, probiotics strengthen the gut barrier by reducing intestinal permeability and systemic inflammation, thereby protecting the brain from inflammatory stress (Rahmannia et al., 2024). Through these mechanisms—such as reducing neuroinflammation, modulating neurotransmitter levels, and influencing neuronal circuitry via the vagus nerve—probiotics contribute to a more favorable biochemical environment for brain health and emotional regulation.

### 2.2 Clinical evidence and limitations

A review by Dronkers et al. (2020) reported that probiotics (the most studied strains were *Lacticaseibacillus rhamnosus* GG (LGG) and *Bifidobacterium animalis* subsp. *lactis* BB12) were administered in over 1,000 clinical trials with an average sample size of 74 participants. These clinical studies addressed 700 different diseases and conditions and were registered at ClinicalTrials.gov run by the United States National Library of Medicine and/or the World Health Organization’s International Clinical Trials Registry Platform (Dronkers et al., 2020). In addition to strain and dosage activity, the potential benefits of probiotics are often limited by interactions with the host microbiome (Suez et al., 2019). In the last 5 years, there have been more than 500 case studies/year, systematic reviews and meta-analyses covering a wide range of conditions, confirming that probiotic interventions have beneficial effects in various gastrointestinal, metabolic, immunological, neuropsychiatric and various other conditions (Bagdadi et al., 2025). In particular, probiotics have been shown to exert condition-specific benefits through multiple mechanisms. In psychiatric disorders (excluding schizophrenia), clinical studies reported improvements in mood regulation, anxiety reduction, and cognitive performance, possibly mediated by modulation of the gut-brain axis and reduction of systemic inflammation (Hong et al., 2022; de Lima et al., 2025). In allergic diseases, probiotics were associated with decreased symptom severity and improved immunological tolerance, potentially via restoration of gut microbial balance and enhancement of regulatory T-cell responses (Xi et al., 2025). For patients with type 2 diabetes mellitus, probiotic supplementation improved glycemic control, insulin sensitivity, and inflammatory markers, highlighting their role in metabolic regulation (Wang et al., 2024). In gastrointestinal disorders such as irritable bowel syndrome, probiotics alleviated abdominal pain, bloating, and irregular bowel habits, likely through normalization of gut motility and modulation of the gut microbiota (Ceccherini et al., 2022; Li et al., 2020). Similarly, in inflammatory bowel diseases, osteoarthritis, and chronic kidney disease, clinical evidence supports their role in reducing disease activity, inflammatory biomarkers, and oxidative stress, thereby contributing to improved quality of life (Karim, 2025; Liu C, et al., 2024). However, for diseases such as COVID-19, systemic sclerosis, Crohn’s disease and scleroderma, the results of the studies are inadequate. Clinical efficacy remains limited, and interpretation of results is compromised by the lack of standardization when using different strains (even within the same species), the inability to determine individual contributions in multi-strain formulations and the lack of consistency in dosage and duration of probiotic use. Furthermore, reproducibility of clinical trials is limited as they differ in design (small numbers of participants and heterogeneous groups, short duration), do not capture participants’ health status, age, diet and baseline microbiota, focus on short-term symptom improvement and report symptom relief without investigating mechanisms, e.g., metabolomics and immunomodulation. Common probiotic strains have been extensively studied and are well known for their health benefits (Table 1).

**Table 1:** Documented health benefits of specific probiotic strains from clinical and preclinical studies (covered period 2020–2025).

Group	Strain	Health benefits	References
*Lactobacillus*	*L. rhamnosus GG (LGG)*	Well documented survival in gastric disorders; reduces diarrhea and improves the integrity of the gut barrier. Produces GABA and modulates emotional behavior via the vagus nerve in animals. Inhibits pathogens such as *Salmonella* species. Promotes healing of the gut barrier and reduces its permeability. Early LGG colonization inhibits the formation of intestinal tumor in animals. Reduces the risk of allergies.	Bravo et al. (2011); Capurso (2019); Liu et al. (2022b)
	*L. acidophilus*	Inhibits pathogens such as *Salmonella* and *C. perfringens*; improves lactose digestion, immune response and cholesterol levels in humans and animal models. Improves the balance of the gut microbiota, reduces symptoms such as bloating and abdominal discomfort and shortens transit time of food. Reduces cardiovascular risk by lowering systolic and diastolic blood pressure, LDL cholesterol and triglycerides, and improving overall lipid profiles. Improves type 2 diabetes by improving intestinal barrier function, suppressing inflammatory responses in the liver and colon and regulating glucose and lipid metabolism in the liver.	Ejtahed et al. (2011); Lekić (2024); Liu Y, et al. (2024); Yan et al. (2019)
	*L. plantarum*	Improves some autism symptoms (hyperactivity/ impulsivity, disruptive and disorderly behavior) suggesting involvement in neurobehavioral regulation. Recommended for the treatment of diabetes; modulates inflammatory responses, inhibits enzymes involved in glucose metabolism, improves insulin sensitivity, restores gut microbiota, and produces short-chain fatty acids. Lowers LDL and total cholesterol levels. Improves gastroenterological health, including reducing abdominal pain and regulating the composition of intestinal microbiota. Improves periodontal health, including reduced pocket depth and bleeding on probing.	Aljohani et al. (2024); Kerlikowsky et al. (2025); Kumar et al. (2025); Liu et al. (2019)
	*Li. reuteri*	Improves adaptive behavior and social interaction in autism patients. Potential therapy for infantile colic and a supportive strategy for diarrhea, constipation and *H. pylori* infection. Relieves abdominal pain, improves symptoms of inflammatory bowel disease, diverticulitis, colon cancer and liver diseases. Reduces the production of pro-inflammatory cytokines and promotes the development and function of regulatory T-cells, thereby alleviating inflammatory diseases.	Mazzone et al. (2024); Mu et al. (2018); Peng et al. (2023); Schmitt et al. (2023)
	*L. paracasei*	Prevents stress-irelated metabolic disorders. Beneficial effects on anxiety-like behavior. Reduces serum LDL cholesterol in adults with hypercholesterolaemia. Modulates lung immunity leading to the improvement in influenza infection and alleviating respiratory infections.	Belkacem et al. (2017); Karen et al. (2021); Khongrum et al. (2023)
*Bifidobacterium*	*B. lactis*	Supports intestinal motility, improves barrier function and reduces inflammation and gut transit time. The combination of *B. lactis* with phototherapy improves the elimination of jaundice by increasing the number of beneficial gut bacteria, thusfacilitating the recovery of newborns. It can be associated with the reduction of body fat through changes in metabolic health parameters (serum triglyceride and adipokine levels).	Bravo et al. (2011); Cheng et al. (2021); Lee et al. (2024); Tsai et al. (2025)
	*B. longum*	Improves depression and quality of life in patients with irritable bowel syndrome; reduces cortisol levels and improves sleep in healthy adults; modulates brain activity to increase stress resistance. Prevents and alleviates various digestive diseases, by maintaining gut homeostasis by repairing the intestinal mucosal barrier, stimulating Paneth cell activity, and modulating the composition of the gut microbiota. Lowers total and LDL cholesterol levels and reduces obesity-related markers.	Allen et al. (2016); Kim et al. (2022); Pinto-Sanchez et al. (2017); Xiao et al. (2025)
*Saccharomyces*	*S. boulardii*	Prevents acute and antibiotic-associated diarrhea; supports *H. pylori* therapy; reduces gut motility and modulates the microbiota to reduce inflammation. Improves pain sensation, inflammatory and oxidative stress biomarkers in patients with knee osteoarthritis.	Bustos Fernández et al. (2023); Dolatkhah et al. (2024); Gu et al. (2022)

### 2.3 Innovations

Recent innovations in the use of probiotics include the development of multi-strain and customized *psychobiotic* formulations aimed at influencing anxiety and depression, cognition and neuroinflammation (Kamal et al., 2025; Messaoudi et al., 2011); the combination of probiotics with prebiotics (fiber-based substrates that promote the growth of probiotics), resulting in *synbiotic* therapies for enhanced effects on the gut and brain; next-generation encapsulation and delivery technologies (e.g., microbiotics); and the development of new products (e.g., microencapsulation, enteric coatings, phage-probiotic combinations) to improve strain viability and targeted release in the gut (D’Amico et al., 2025; Ranadheera et al., 2017); precision approaches using specific strains such as *Limosilactobacillus reuteri* (*L. reuteri*) for behavioral modulation in autism or *B. longum* for stress resistance; and ongoing research on genetically engineered probiotics that produce neuroactive compounds (e.g., GABA, serotonin) or immunomodulatory molecules directly in the gut (Charbonneau et al., 2020). Taken together, these innovations are moving probiotics from general gut health agents to targeted therapeutics for gut- and brain-related conditions such as anxiety, depression, Alzheimer’s, Parkinson’s, and autism spectrum disorders. They are increasingly recognized as modulators of gut and systemic health, including the gut-brain axis (GBA; Ali et al., 2025). Future studies should conduct longer-term studies considering the standardized strain-specific design and protocols, and personalized approaches based on patients’ microbiome profiles.

## 3 Prebiotics

Prebiotics have long been used to modulate gut microbiota metabolic function, and enhance host health (Sanders et al., 2019). Microbial enzymes and substrates could provide important potential for prebiotics production, with extensive use in the food and pharmaceutical industries. Among the most extensively employed prebiotics are galacto-oligosaccharides (GOS), xylo-oligosaccharides (XOS), and fructo-oligosaccharides (FOS).

### 3.1 Galacto-oligosaccharides

Several innovative microbial approaches have been extensively explored for the production of galacto-oligosaccharides (GOS), offering sustainable and efficient alternatives to conventional synthesis methods. One remarkable process includes the *β*-galactosidase immobilized on different substrates (Hackenhaar et al., 2021; Carević et al., 2018). Geiger et al. (2016) stated that a simple recombinant β-galactosidase from *Streptococcus thermophilus* DSM 20259 could convert 80% of whey lactose to GOS in 5 h. They also reported that ≈ 1 kg of GOS was produced from 3 kg of whey permeate powder. From Greek yogurt, the β-galactosidase synthesized by *Cryptococcus laurentii* whole-cells produced the GOS whey at 36% (w/w), and 50% of the initial lactose was converted at a specific productivity equal to 2.3 mg/U·h (Fischer and Kleinschmidt, 2021). Based on an expression system developed on the T7 RNA polymerase promoter in *E. coli*, Kittibunchakul et al. (2019) noted a high recombinant β-galactosidase activity (26,000 U/L). This value was 28-fold and 1,000-fold higher than the production of native β-galactosidase from *L. helveticus* DSM 20075 when grown on lactose and glucose, respectively. To improve their catalytic activity and GOS production, seven β-glucosidase mutants were obtained from *Thermotoga naphthophila* RKU-10. Interestingly, the F414S mutant showed efficient properties since the GOS production was improved from 140 mM to 207 Mm using 0.2 mM lactose (Yang et al., 2018). On free or immobilized forms, commercial enzymes have been employed in GOS production. The primary commercial enzymes used in GOS synthesis process are bacterial β-galactosidases (e.g., *Kluyveromyces lactis*) and fungal (e.g., *Aspergillus oryzae*) sources (Maráz et al., 2022). From different microbial sources, Mano et al. (2019) confirmed that *Kluyveromyces lactis* commercial enzyme named Lactozyme™ 2,600 L could display an optimal performance for lactose conversion, yield and specific productivity (50 g GOS/g enzyme×h).

### 3.2 Xylo-oligosaccharides

Due to their superior properties compared to other prebiotics, xylo-oligosaccharides (XOS) have gained more attention. XOS are stable across a broad pH range (2.5–8.0) and at temperatures up to 100 °C. Moreover, xylobiose has 0.3–0.4 times the sweetness of sucrose, and the approved daily dietary intake of XOS (2.1 g) is lower than that of most other oligosaccharides (Amorim et al., 2019). Notwithstanding these merits, XOS is more costly than the other prebiotics (Yegin, 2023). Because of the diverse enzyme systems they possess, each microorganism has a distinct XOS utilization pattern. For instance, XOS containing uronic acid are utilized by only a few bifidobacteria of human origin. *Bifidobacterium adolescentis* can metabolize both arabino-XOS and linear XOS (Falck et al., 2013), whereas *Levilactobacillus brevis* grows preferentially on linear XOS (Precup et al., 2022).

To date, most commercial xylanases are produced by bacteria (e.g., *Bacillus* and *Streptomyces*) and fungi (e.g., *Thermomyces*, *Trichoderma*, and *Aspergillus*). As a result, xylanases from these sources became dominant in the enzyme market. To categorize the extracellular enzymes including xylanases synthesized by *Bacillus* sp. AR03, Hero et al. (2021) exploited a proteomic approach. By LC–MS/MS identification, these authors reported a glucuronoxylanase GH30-8 and an endoglucanase GH5-2. From *Bacillus* sp. strain BP-7. Gallardo et al. (2010) reported that GH5 xylanase (Xyn5B) was able to act on linear XOS, and xylooligomers with methylglucuronic groups were generated. A novel xylanase produced by *Streptomyces* spp. (B6) was able to generate two xylanases attributed to GH10 and GH11 families (Liu et al., 2022a). In another study conducted by Boonchuay et al. (2014), *Streptomyces thermovulgaris* TISTR1948 used xylanase for XOS production. The enzyme production was conducted at 50 °C and 250 rpm for 96 h utilizing the rice straw as a carbon source, and the principal oligomer was xylobiose with 85.15 mg/g. Adsul et al. (2009) described an efficient process for the hydrolysis of xylan by using *Streptomyces matensis* xylanase, yielding mainly xylotriose and xylobiose as the predominant XOS products. Enzymes from *Aspergillus* have also been employed for XOS production using lignocellulosic biomass. For example, Akpinar et al. (2007) applied a two-stage approach to produce XOS from cotton stalk, first extracting xylan with KOH and then hydrolyzing it with commercial *Aspergillus niger* xylanase. Maximum XOS production was observed at 40 °C and pH 5.4 with 2% xylan (10 mL) and 4.4 U/mL enzyme (1 mL). To produce XOS by a packed bed reactor in continuous mode, Aragon et al. (2013) employed xylanase immobilization of *Aspergillus versicolor* by using several support materials. The glyoxyl agarose constitutes the most effective support for xylanase immobilization and maintained till 85% of its catalytic activity. After incubation at 60 °C, the immobilized enzyme was nearly 700 times more stable than the free fraction, and retained full activity after 10 cycles of 1-h process. In addition, the immobilized xylanase delivered 2.5-fold higher xylobiose production as compared to free fraction.

The xylan bioconversion from agricultural residues into XOS without prior pre-treatment has been revealed to be possibly economical alternate for industrial application. Crude xylanase from *Aspergillus fumigatus* R1 was qualified to yield 1.08% (w/w) of XOS from raw wheat husk xylan. XOS with a DP up to 5 were detected in the final hydrolysate, being xylobiose the greatest principal oligosaccharide during the entire reaction time (Chavan et al., 2023). This enzymatic process avoids the formation of unwanted by-products typical of chemical extraction.

### 3.3 Fructo-oligosaccharides

Fructo-oligosaccharides (FOS) are enzymatically produced from sucrose through a transfructosylating reaction catalyzed by *β*-fructofuranosidase (FFase) or fructosyltransferase (FTase) enzymes. *Aspergillus flavus* NFCCI 2364 FTase was explored to produce FOS from 16 agro-wastes (Ganaie et al., 2017). Smaali et al. (2012) used *β*- Ffase from *Aspergillus awamori* NBRC4033, demonstrating high yields and indicating the efficiency of agro-residue biomasses as substrates. Silva et al. (2013) stated that inulinase from *Aspergillus niger* (*A. niger*) and *Kluyveromyces marxianus* (*K. marxianus*) NRRL Y 7571 was able to generate FOS from inulin, with specific yields of kestose, nystose, and fructosyl nystose. Diez-Municio et al. (2013) showed that the inulosucrase from *Lactobacillus gasseri* DSM 20604 could generate FOS and maltosylfructosides (MFOS) from sucrose and sucrose/maltose mixtures. For short-chain FOS (scFOS) and oligolevans production, an inventive two-phase system of levansucrase (from *Bacillus amyloliquefaciens*)/endo-inulinase (from *A. niger*) used the sucrose. This system permitted levansucrase to create levans, while endoinulinase monitored molecule size, with 6-kestose being the primary scFOS (Ni et al., 2021). In addition, the immobilization of levansucrase improved the levan production over scFOS (Ni et al., 2021). To produce FOS from sucrose, Soliman et al. (2017) studied the immobilization of inulinase isolated from *A. niger*. The used material was on polyurethane foam, attaining a 30% total FOS yield, including GF2, GF3, and GF4. Bersaneti et al. (2018) revealed that levansucrase from *B. subtilis* could generate FOS and levan concurrently at 41 g/L of FOS and 87 g/L, respectively. Huang et al. (2016) stated that *Aspergillus aculeatus* M105 produced extracellular FTase, achieving FOS yields of 68 and 66% (w/w), respectively. By using the inulosucrase (IslA4), issued from *Leuconostoc citreum*, and sucrose, Peña-Cardeña et al. (2015) produced FOS containing compounds f-nystose, nystose, neokestose, 1-kestose and 6-kestose.

### 3.4 Other prebiotics

Many additional produced prebiotics can be isolated or produced from several microbial sources. Manno-oligosaccharides (MOS) production emphasizes a substantial progress in the utilization of renewable resources for producing appreciated prebiotic. A genetically engineered endo-β-(1,4)-mannanase, isolated from *B. subtilis* and expressed in *Escherichia coli*, could generate mannans to MOS with a degree of polymerization arraying from 4 to 7 (Sathitkowitchai et al., 2022). The β-mannanase from *Penicillium aculeatum* APS1 can degrade glucomannan (from konjac) and galactomannan (from locust guar and bean gums). The enzyme produces low molecular weight at DP ≤ 4 (Bangoria et al., 2021). From *Streptomyces cyaenus*, a mannanase hydrolyzed palm cake kernel with oligo-mannans (DP ≤ 7). In addition, the mannotriose and mannobiose were detected throughout the reaction period (up to 8 h; Purnawan et al., 2017).

To improve the production of isomalto-oligosaccharides (IMOs), recombinant enzymes engineering showed an important progression. In this line, Kaulpiboon et al. (2015) used pullulanase, a modified amylomaltase Y101S, and transglucosidase from *A. niger*. This enzyme blend enabled the production of long-chain IMOs at pH 7.0 and 40 °C.

Table 2 summarizes some examples of major microbial enzymatic producing prebiotic (GOS, XOS and FOS). Besides the biochemical and production aspects described above, the physiological and clinical effects of prebiotics have also been investigated extensively. Several studies have shown that the intake of prebiotics such as GOS, FOS, and XOS can positively alter the composition of the gut microbiota, increase short-chain fatty acid production, and improve intestinal barrier function. In clinical trials, prebiotics have been associated with positive results in gastrointestinal health, including relief from constipation and irritable bowel syndrome, with beneficial effects on metabolic disorders such as obesity and type 2 diabetes (Sanders et al., 2019; Markowiak and Śliżewska, 2017; Davani-Davari et al., 2019; Gibson et al., 2017). These evidences highlight the importance of prebiotics not only as industrially significant compounds but also as key regulators of human health.

**Table 2:** Examples of major enzymatic produced prebiotic (GOS, XOS and FOS), including microorganisms, enzyme sources, processes and yields.

Produced prebiotic	Process	Yield	References
Galacto-oligosaccharides (GOS)—β-Galactosidase microbial origin			
*Lactobacillus acidophilus* ATCC 4356	Immobilization of β-Galactosidase (resins)	17.13% (90 g/L)	Carević et al. (2018)
*L. delbrueckii* subsp. *bulgaricus* strain 43	β-Galactosidase	34% (70.91 g/L)	Arsov et al. (2022)
*B. circulans*	Immobilization of β-Galactosidase (chitosan beads)	40% (159.4 g/L)	Hackenhaar et al. (2021)
*Lactiplantibacillus plantarum* WCFS1	Immobilization of β-Galactosidase (*Lactobacillus* Cell Surface)	32% (205 g/L)	Pham et al. (2019)
*L. helveticus* DSM 20075	Immobilization of β- Galactosidase (HisTrap HP Ni)	32% (155 m g/L)	Kittibunchakul et al. (2019)
*Limosilactobacillus reuteri* L103 and *Lactobacillus bulgaricus* DSM 20081	Immobilization of β- Galactosidase (chitin beads)	>91%	Pham et al. (2017)
*Pseudozyma tsukubaensis* and *Pichia kluyveri*	Fermentation using whole living cells at 30 °C, pH 7.0 and 24 h	14 and 15%, (2.62 and 2.34) g/L/h, respectively	Fai et al. (2014)
Commercial	β-galactosidase from *Bacillus circulans*	37% (290 μmol/g)	Usvalampi et al. (2018)
*Aspergillus oryzae*	Immobilization of β-Galactosidase (3 functionalized -modified glass beads)	39.3%	Eskandarloo and Abbaspourrad (2018)
*Bacillus circulans*	Immobilization of β- Galactosidase (*Microporous Polyvinylidene* fluoride membrane)	28%	Palai and Bhattacharya (2013)
*Bacillus circulans*	Immobilization of β- Galactosidase (*Microporous Polyvinylidene* fluoride membrane)	30%	Palai et al. (2014)
*Aspergillus oryzae*	Immobilization of β- Galactosidase (Sol–gel carriers)	26% (8.7 g/Lh)	Jovanovic-Malinovska et al. (2012)
*Aspergillus oryzae*	Immobilization of β- Galactosidase (Polyvinyl alcohol (PVA) lenses)	31% (31 g/Lh)	Jovanovic-Malinovska et al. (2012)
*Aspergillus oryzae*	Immobilization of β- Galactosidase (Polyvinyl alcohol (PVA) lenses)	23–30% (65–117 g/Lh)	Jovanovic-Malinovska et al. (2012)
*Aspergillus oryzae*	Immobilization of β- Galactosidase (Polyvinyl alcohol (PVA) lenses)	17–25% (65–117 g/Lh)	Jovanovic-Malinovska et al. (2012)
*Thermotoga naphthophila* RKU-10	β-Galactosidase (at pH 6.5 and 75 °C with 100%)	23.28 g/Lh	Yang et al. (2018)
*Lactococcus lactis*	β-Galactosidase	55% (197 g/L)	Yu and O’sullivan (2014)
*Aspergillus oryzae*	Fermentation by using 50% (w/w of) lactose monohydrate	28 g/L	Vera et al. (2012)
Xylo-oligosaccharides XOS—xylanase microbial production			
Xylanase secreted by *Pichia stipitis*	Fermentation by using at pH 5.8 and temperature 44 °C with 5.73 U of xylanase enzyme incubation for 17.5 h	9.55 g/100 g xylan	Samanta et al. (2016)
Xylanase secreted by *Pichia stipitis*	Fermentation by using pH 5.4, 50 °C with orbital shaking at 150 rpm at 12 h, and	31.8% (5.29 g/L)	Bian et al. (2013)
Commercial β-D-xylanase	10 U/mL at 50 °C for 24 h.	70.6%	Peng et al. (2011)
crude xylanase	Concentration: 4.5%, pH, 5.5, T° 55 °C and 18 h	24% (2.37 mg/mL)	Jnawali et al. (2018)
Endoxylanase from *Trichoderma viride*	Concentration 2.65 U, T = 40 °C, pH = 4 at 8 h.	37% (5.7 mg/mL)	Jayapal et al. (2013)
*Aspergillus foetidus* MTCC 4898	1% xylan, 20 U, 45 °C, 8 h	673 mg/g xylan	Chapla et al. (2012)
*Aspergillus versicolor*	18 mg/mL xylan, 28 U/g xylan, 25 °C, pH 5.0, 7 h	180 mg/g xylan	Aragon et al. (2013)
*Aspergillus fumigatus* M51	7% xylan, 120 U/g xylan, 50 °C, pH 5.5, 130 rpm, 24 h	271 mg/g xylan	de Figueiredo et al. (2017)
*Aspergillus niger* BCC14405	1 mg/mL xylan,10 mg/g xylan, 45 °C, pH 6.0, 24 h	708 mg/g xylan	Aiewviriyasakul et al. (2021)
*Trichoderma viride*	1% xylan, 80 U/g xylanb, 40 °C, pH 5.0, 24 h	175 mg/g xylan	Sabiha-Hanim et al. (2011)
*Trichoderma viride*	2% xylan,12 U/g xylan, 50 °C, pH 5.0, 200 rpm, 6 h (Fed batch strategy at 7 h)	Batch: 270 mg/g xylan Fed batch: 670 mg/g xylan	Qian et al. (2020)
*Trichoderma reesei*	3 U/mL, 50 °C, pH 4.8, 150 rpm, 48 h	446 mg/g xylan	Su et al. (2021)
*Trichoderma viride*	2.2% xylanb, 13.25 U/g xylan, 55 °C, pH 5.0, 100 rpm, 12 h	229 mg/g xylan	Rathamat et al. (2021)
*Thermomyces lanuginosusis*	10 U/g xylan, 50 °C, pH 6.5, 70 rpm, 36 h	82 mg/g biomass	Singh et al. (2019)
*Thermoascus aurantiacus* IMI 216,529	2% xylan, 5 U/g xylan, 50 °C, 20 h	25 mg/g xylan	Katapodis et al. (2002)
*Thermoascus aurantiacus* ATCC 204,492	2.6% xylan, 60 U/g xylan, 50 °C, pH 5.0, 150 rpm, 96 h	371 mg/g xylan	Brienzo et al. (2010)
*Aureobasidium pullulans* CCT 1261	Microbial bioprocess: 28 °C, pH 5.0, 150 rpm, 12 h	168 mg/g xylan	Gautério et al. (2018)
*Aureobasidium pullulans* NRRL Y-2311–1	7.2% xylan, 240 U/g xylan, 40 °C, pH 5.0, 100 rpm, 48 h	312 mg/g xylan	Surek et al. (2021)
*Paecilomyces variotii* NRRL 1115	1% xylan, 55 °C, pH 5.0, 6 h	-	Abdella et al. (2021)
*Bacillus aerophilus* KGJ2	5% xylan, 20 U/g xylan, 70 °C, pH 4.0, 12 h	114 mg/g xylan	Gowdhaman and Ponnusami (2015)
*Bacillus mojavensis* UEB-FK	2% xylan, 12 U/g xylan, 50 °C, pH 4.0, 100 rpm, 8 h	290 mg/g xylan	Kallel et al. (2015)
*Bacillus subtilis* KCX006	30%, 37 °C, pH 7.0, 72 h	48 mg/g biomass	Reddy and Krishnan (2016)
*Bacillus subtilis* CCT 7611	1% (108 CFU/mL), 37 °C,125 rpm, 48 h	65 mg/g biomass	Reque et al. (2019)
*Streptomyces rameus* L2001	2% xylan, 4 U/mL, 50 °C, pH 7.0, 140 rpm, 2 h	150 mg/g corn cob xylan 105 mg/ g bean culms xylan	Li et al. (2012)
*Streptomyces thermovulgaris* TISTR1948	10% biomass, 100 U/g biomass, 55 °C, pH 6.5, 18 h	35.6 mg/g biomass	Seesuriyachan et al. (2017)
*Streptomyces* sp. B6	200 U/mL, 50 °C, pH 8.0, 24 h	390 mg/g substrate	Liu et al. (2020)
*Clostridium* sp. BOH3	5% xylan, 20 U/g xylan, 50 °C, pH 5.0, 12 h	572 mg/g	Rajagopalan et al. (2017)
*Paenibacillus barengoltzii*	50 U/mL, 50 °C, pH 6.5, 150 rpm, 12 h	750 mg/g xylan	Liu et al. (2018)
*Pichia stipitis*	2% xylan, 25 U/g xylan, 50 °C, pH 5.4, 14 h	368 mg/g	Yang et al. (2011)
*Pichia stipitis*	2% xylan, 25 U/g xylan, 50 °C, pH 5.4, 150 rpm, 12 h	318 mg/g xylan	Bian et al. (2013)
Fructo-oligosaccharides (FOS)			
*Aspergillus niger* and *K. marxianus* NRRL Y-7571	Inulinases immobilization (glutaraldehyde)	26.62 and 30.62%, respectively	Silva et al. (2013)
*Penicillium*	beta—fructofuranosidase: 25.5 °C and 67.8 h.	58.7 g/L	Nascimento et al. (2016)
*Aspergillus awamori* NBRC 403	β-Dfructofuranosidase immobilization (chitosan and glutaraldehyde)	55% (121.5 g/L)	Smaali et al. (2012)

## 4 Synbiotics

The term Synbiotic, first defined in 1995 by Gibson and Roberfroid to refer to a combination of probiotics and prebiotics, describes a combination in which prebiotics enhance the activity of probiotics (Dahiya and Nigam, 2022; Ashique et al., 2024). At a meeting of the International Scientific Association for Probiotics and Prebiotics (ISAPP) in 2019, this phrase was revised to a mixture containing live microorganisms and substrate(s) that are selectively utilized by the host microorganisms and provide a health benefit to the host (Gibson et al., 2017; Swanson et al., 2020; Dahiya and Nigam, 2022). Synbiotics are classified into two forms. Complementary synbiotics consist of a combination of probiotic and prebiotic components that each independently meet minimum efficacy criteria; these components act independently to support host health. In comparison, synergistic synbiotics are defined as a system in which the coordinated interaction of selected probiotic microorganism strains and prebiotic substrates that specifically promote the growth and activity of these strains work together to provide targeted physiological benefits (Kleerebezem and Führen, 2024). Mechanically, synbiotics represent the combination of the biochemical properties of prebiotics and the functional effects of probiotics. While prebiotics selectively stimulate the growth of beneficial microbes, the presence of specific probiotic strains ensures direct interaction with the host. This synergistic interaction provides both metabolic and immunological benefits and creates a more comprehensive effect than either component alone (Swanson et al., 2020; Kolida and Gibson, 2011).

Studies show that synbiotic intake contributes to the alleviation of irritable bowel syndrome, metabolic syndrome, inflammatory bowel diseases, diarrhea and skin problems such as atopic dermatitis (Markowiak and Śliżewska, 2017). Wang et al. (2024), investigated whether the symptoms of Autism Spectrum Disorder (ASD) could be improved through modulation of gut microbiota. They treated mouse models with the disorder with a daily synbiotic treatment consisting of a combination of *Limosilactobacillus reuteri* (*L. reuteri*) and inulin for 4 weeks. They reported that this combination alleviated the social impairments associated with ASD, in part through its regulatory effects on the gut-brain axis (Wang et al., 2024). Palepu et al. (2024) investigated the effect of *Faecalibacterium prausnitzii* (ATCC-27766) in combination with fructo-oligosaccharides (FOS) and galacto-oligosaccharides (GOS) in 2024 treatment-resistant depression (TRD) rat models and suggested that this synbiotic may reverse TRD-like symptoms in rats by positively affecting gut health, neuroinflammation, neurotransmitters and gut microbial composition. Bonfili et al. (2017), supplemented the diet of 3xTg-AD Alzheimer’s disease mice with a red lentil (prebiotic) based cookie enriched with neuroprotective probiotics and performed behavioral, biochemical and molecular tests. They reported that short-term memory improved after the treatments and that the combined use was successful compared to the individual ingredients. Johnson et al. (2025), investigated the effect of dietary synbiotic supplementation on behavioral, neurochemical and microbial parameters in W36 Hy-Line laying hens, focusing on modulation of the microbiota-gut-brain (MGB) axis. Over a 10-week intervention, they found that in chickens receiving a synbiotic diet, agonistic behaviors, including pecking and fighting, were significantly reduced, while dopamine and serotonin levels were increased. They stated that MGB axis regulation contributed to improved welfare and reduced stress-induced behaviors. Ghorbani et al. (2018) aimed to evaluate the efficacy of a 6-week synbiotic supplement in the treatment of moderate depression and gave fluoxetine (20 mg/day) to all patients for 4 weeks. They then added a synbiotic capsule (plus fluoxetine) or placebo (plus fluoxetine) to the treatment for 6 weeks.

And they found the efficacy of the synbiotic as an adjuvant treatment for moderate depression. Shinde et al. (2020), wanted to develop dietary strategies to help address the increasing cases of inflammatory bowel diseases (IBD) and investigated the efficacy of green banana resistant starch (GBRS) and probiotic *Bacillus coagulans* (*B. coagulans*) MTCC5856 spores for improving dextran-sulfate sodium (DSS)-induced colitis in mice. They found that synbiotic supplementation with *B. coagulans* and GBRS improved the overall inflammatory status of the experimental IBD model through synergistic functioning. They recommended investigating the effect of this practice in reducing inflammation in human IBD. Polakowski et al. (2019), investigated the effect of preoperative synbiotic administration in colorectal cancer patients with colorectal resection. They randomized patients to receive synbiotics (Simbioflora, Farmoquimica, São Paulo, Brazil) or placebo (maltodextrin) 8 days before surgery. They found that 7 days of preoperative synbiotic use in colorectal cancer patients relieved the inflammatory state and reduced morbidity, length of hospital stay and antibiotic use.

When the above findings are evaluated, it is understood that synbiotics exhibit beneficial effects in various conditions, including gastrointestinal, metabolic, dermatological, and neuropsychiatric disorders. Despite the diversity of tested models and combinations, several mechanisms consistently emerge, such as modulation of the gut-brain axis via neurotransmitter regulation, alleviation of systemic and local inflammation, and improvement of gut microbial balance. However, several limitations must be acknowledged. Most studies are based on animal models, limiting translation to humans. Furthermore, existing clinical studies are generally short-term and have limited sample sizes. Moreover, heterogeneity in strain-substrate combinations and dosages makes direct comparisons between studies difficult and hinders the establishment of standardized protocols. Future research should prioritize conducting large-scale, long-term clinical studies to establish the clinical efficacy and safety of synbiotics.

## 5 Gut microbiota and brain communications

Probiotic research has expanded far beyond gut microbiota balance, revealing their roles in strengthening gut barrier integrity, regulating immune responses, producing bioactive compounds, and interacting directly with host cells, thus broadening their therapeutic potential (Piccioni et al., 2023). While well-established strains include *Lactobacillus*, *Bifidobacterium*, and *Saccharomyces*, emerging candidates such as *Roseburia* spp., *Akkermansia* spp., and *Faecalibacterium* spp. show potential for future applications (Sanders et al., 2019). Many of these functional roles are supported primarily by *in vitro* and animal model data, and while preclinical results are encouraging, translation into clinically validated outcomes remains inconsistent and strain-specific, as not all effects observed in experimental models are confirmed in humans, and even among clinical trials, heterogeneity in study design, dosage, strain selection, and host response complicates definitive conclusions. Additional limitations include the lack of long-term safety data, and potential interactions between strains or with the host that may diminish efficacy, and the need for personalized approaches to optimize benefits. Advances in genome sequencing, microbiota analysis, and real-time *in vivo* sampling are expected to help address these gaps, leading to a clearer understanding of their mechanisms and health benefits (Sanders et al., 2019).

Prebiotics are substances selectively utilized by host microorganisms to promote health benefits, including the modulation of gut microbiota and the production of beneficial metabolites such as short-chain fatty acids (SCFAs) and tryptophan (Galica et al., 2022). Initially known for stimulating the growth of bifidobacteria and lactobacilli, prebiotics are now recognized for their broader effects on metabolic and physiological systems, such as immune modulation, defense against pathogens, improved intestinal function, and enhanced mineral absorption (Piccioni et al., 2023). Common prebiotics, such as fructo-oligosaccharides (FOS), inulin, and resistant starches, are commercially available and contribute to optimizing the human microbial environment. However, while the mechanisms of action have been outlined through *in vitro* and animal models, confirming these effects in humans remains challenging (Sanders et al., 2019).

Synbiotics, a combination of probiotics and prebiotics, support the growth and activity of beneficial gut bacteria, promoting digestive health and strengthening immune function. By enhancing gut microbiota balance, they offer a comprehensive approach to improving overall well-being through dietary supplementation (Al-Habsi et al., 2024).

**Figure 1:**
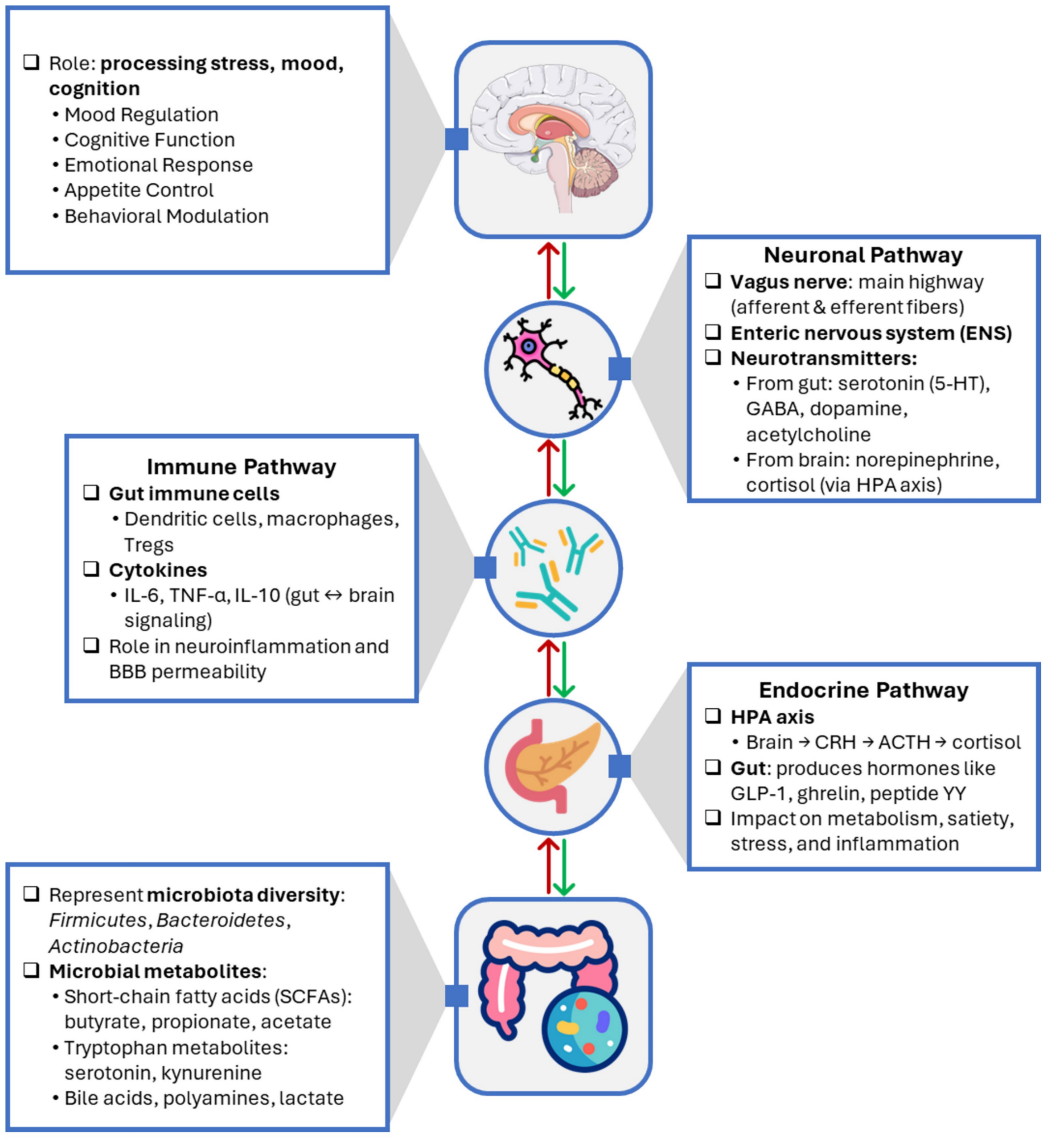
Bidirectional communication pathways of the gut-brain axis.

To clarify the specific effects of probiotics, prebiotics, and synbiotics on gut-brain axis and their mechanisms of action, Table 3 summarizes their health benefits and potential roles in various physiological processes. Figure 1 illustrates the bidirectional communication pathways of the gut-brain axis, showing how neuronal, endocrine, and immune signaling mediate interactions between the gut microbiota and the central nervous system. The gut microbiota modulates brain function through neuronal (vagal and enteric nervous system), endocrine (gut-derived hormones and HPA axis), and immune (cytokines, inflammation) pathways. In return, the brain influences gut physiology through stress, emotion, and autonomic regulation. Microbial metabolites such as SCFAs, neurotransmitters, and tryptophan derivatives act as mediators in this bidirectional dialog.

**Table 3:** Impact of probiotics, prebiotics, and synbiotics on the gut-brain axis.

Nature	Probiotics/Prebiotics/Synbiotics	Health benefit	Potential mechanism	Reference
*Lactiplantibacillus plantarum* 20,174	Probiotics	Improving cognitive function and reducing neurodegeneration	Modulation of the gut-brain axis, restoration of hippocampal amyloid beta, p-tau, α-synuclein, and BDNF levels, and reduction in oxidative stress and inflammation	Shahin et al. (2025)
*Asparagus officinalis* extract	Prebiotics		Restoration of neurotransmitter balance, modulation of gut microbiota composition by increasing *Lactobacillus* species and reducing harmful bacteria	
Combination of *L. plantarum* and *A. ofcinalis*	Synbiotics		Synergistic modulation of gut microbiota, enhanced antioxidant effects, reduced inflammation, and improved neurotransmitter balance, leading to superior cognitive restoration	
Heat-killed lactic acid bacteria (e.g., *Lactococcus lactis*, *Lactobacillus* strains)	Probiotics	Immunomodulation and immune barrier support	Inhibition of nitric oxide production (up to 86.2%) and suppression of lipopolysaccharide-induced nitric oxide synthase and cyclooxygenase-2 expression	Kang et al. (2021)
*Lactiplantibacillus plantarum* OLL2712	Probiotics	Protection against memory decline in older adults	Modulation of neuroinflammatory responses, increased IL-10 production, and changes in gut microbiota composition	Sakurai et al. (2022)
Asparagus-derived fructans	Prebiotics	Gut microbiota balance and gut health improvement	Stimulation of beneficial bacterial growth, microbial fermentation, production of SCFAs, enhanced microbial metabolism, and antioxidant activity	Hamdi et al. (2023)
*L. acidophilus*	Probiotics	Improved kidney health in diabetic rats, enhanced glycemic control, and increased insulin sensitivity	Modulation of gut microbiota, enhancement of microbial diversity, restoration of the Firmicutes/Bacteroidetes ratio, and reduction of oxidative stress, inflammation, fibrosis, and DNA damage	Al-Ghamdi (2025)
Yogurt (contains live bacteria)	Probiotics	Improvement in gut microbiota and lipid profile in diabetic rats	Increased *Bifidobacterium* and *Lactobacillus* counts	Khalil et al. (2021)
Gum Arabic (dietary fiber)	Prebiotics	Enhancement of gut microbiota, blood glucose regulation, and kidney function	Promotion of beneficial bacterial growth and metabolism	
Combination of yogurt and gum Arabic	Synbiotics	Synergistic improvement in gut microbiota, lipid profile, glucose regulation, and kidney function	Combined effects of probiotics and prebiotics, enhancing microbial balance and metabolic health	
*Limosilactobacillus reuteri* + *Bifidobacterium longum*	Probiotics	Improved gut microbiota composition; increased *Lactobacillus* abundance	Enhanced gastrointestinal resistance; modulation of microbiota to favor beneficial bacteria	Duque et al. (2021)
Galacto-oligosaccharide (GOS)	Prebiotics	Increased *Bifidobacterium* abundance; decreased *Lachnoclostridium* abundance	Promotion of beneficial bacteria; reduction of harmful bacteria; increased production of SCFAs.	
Combination of *L. reuteri* + *B. longum* + GOS	Synbiotics	Positive modulation of gut microbiota and metabolism; increased SCFA concentrations	Synergistic effects on microbiota balance and metabolism; improved microbial fermentation and reduced ammonium levels	
*L. acidophilus*	Probiotics	Regulation of glucose and lipid metabolism, reduction of obesity markers	Modulation of gut microbiota, activation of PPARα for lipid metabolism, reduction of inflammation via TGF-β1 expression	Rangel-Torres et al. (2024)
Inulin	Prebiotics	Reduction of fat mass, improvement of metabolic parameters	Promotion of beneficial gut bacteria, regulation of lipid metabolism, enhancement of gut barrier function	
*L. acidophilus* + Inulin	Synbiotics	Improvement of biochemical markers, reduction of metabolic disturbances	Enhancement of microbial balance, regulation of lipid metabolism genes, reduction of inflammation	
Combined intake of prebiotic and probiotic foods (raw and fermented vegetables)	Synbiotics	Modulation of anxiety symptoms	Interaction between gut microbiota and the gut-brain axis, affecting mood regulation	Tae and Kim (2024)
Dairy-rich diet, Probiotic supplementation	Probiotics	Reduction in depressive symptoms, no significant effect on schizophrenia, stress, and anxiety	Gut-brain axis modulation, neurotransmitter regulation, reduction in inflammation, modulation of central nervous system function	Zagórska et al. (2020)

BDNF, Brain-derived neurotrophic factor; SCFAs, Short-chain fatty acids; GOS, Galacto-oligosaccharide.

### 5.1 Effects on gut microbiota

#### 5.1.1 Mechanisms of action

**Figure 2:**
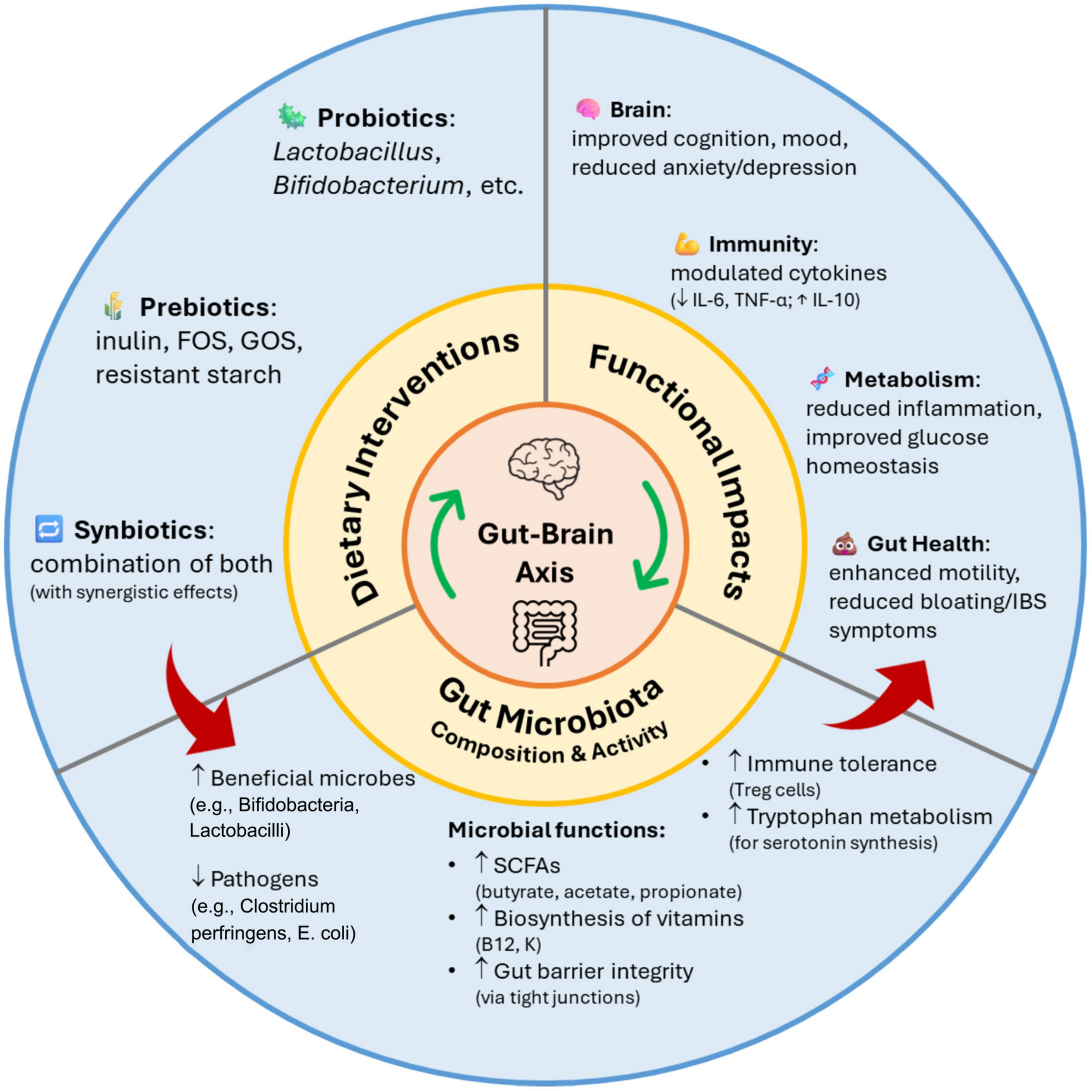
Impact of probiotics, prebiotics, and synbiotics on gut microbiota composition and function.

Probiotic strains influence gut microbiota through nutrient competition, antagonism, cross-feeding, and stability support. *Lactobacillus* and *Bifidobacterium* species produce lactic and acetic acids as key metabolites of carbohydrate fermentation, which lower luminal pH and inhibit pathogen growth. SCFAs, such as acetate, butyrate, and propionate, are generated through the colonic fermentation of dietary fiber and resistant starch. These SCFAs play a crucial role in anti-inflammatory pathways and signaling across various organs, contributing to overall host health (Markowiak-Kopeć and Śliżewska, 2020). Figure 2 exemplifies the modulatory effects of probiotics, prebiotics, and synbiotics on gut microbiota composition and function, highlighting their downstream impacts on gut health, immune response, metabolic regulation, and neurocognitive outcomes. These dietary interventions promote the growth of beneficial microbes while suppressing harmful species, enhancing short-chain fatty acid production, gut barrier integrity, immune tolerance, and neurotransmitter synthesis. The resulting microbial balance contributes to improvements in gut health, immune regulation, metabolic homeostasis, and cognitive and emotional function.

#### 5.1.2 Key microbial metabolites and strain-spesific effects

Probiotic supplementation with *Lactobacillus acidophilus*, *Lactiplantibacillus plantarum*, *Lacticaseibacillus rhamnosus*, and *Enterococcus faecium* for 3 weeks resulted in colonization in both the luminal and mucosal compartments of the colon, increasing lactate levels in the proximal and distal regions. This promoted the growth of lactate-consuming bacteria, enhancing SCFA production, particularly butyrate (Moens et al., 2019). *Lactiplantibacillus plantarum* strains, commonly found in meat, dairy, fruits, and vegetables, are known to support gut microbiota, modulate immune function, lower blood cholesterol, and reduce cancer risk (Zare et al., 2024). Additionally, *L. acidophilus* supplementation improved kidney health in diabetic rats by enhancing gut microbiota diversity and restoring the Firmicutes/Bacteroidetes ratio. This led to better glycemic control, increased insulin sensitivity, reduced oxidative stress, and improved kidney structure with less inflammation and fibrosis, suggesting its potential in mitigating diabetes-related renal complications (Al-Ghamdi, 2025).

Many probiotics exert antagonistic effects by producing organic acids and bacteriocins through saccharolytic metabolism. These bacteriocins help inhibit pathogenic bacteria in the intestines and urinary tract while also contributing to gut homeostasis (Ballan et al., 2020).

#### 5.1.3 Prebiotics and their impact

Prebiotics, such as inulin and polyphenols, are selectively fermented by gut microbiota, generating SCFAs and other beneficial metabolites. These compounds help lower intestinal pH, suppress pathogen growth, and enhance mineral and vitamin absorption (Ballan et al., 2020). Asparagus-derived fructans, similar to commercial fructans, promote beneficial bacterial growth (Hamdi et al., 2023). Despite having a lower degree of polymerization (DP up to 25), they undergo microbial fermentation, supporting gut balance and SCFA production. Additionally, their rich protein and phenolic content may enhance microbial metabolism and antioxidant activity, further benefiting gut health. Phenolic compounds also function as prebiotics by selectively modulating gut microbiota composition through dual mechanisms. They stimulate the growth of beneficial bacteria such as *Lactobacillus*, *Bifidobacterium*, and *Akkermansia*, enhancing the production of SCFAs, which support gut health. Simultaneously, they exert antimicrobial effects by disrupting the structural integrity of pathogenic bacteria (e.g., *Clostridium*, *Staphylococcus*, and *Escherichia coli*; Chiu et al., 2021). This occurs through inhibition of key bacterial proteins (PBP2 and PBP4), impairing peptidoglycan cross-linking, and altering membrane function via proton donation and H^+^-ATPase depletion, thereby creating an unfavorable environment for harmful microbes (Cano et al., 2024). The relationship between polyphenols and gut microbiota is bidirectional, as gut microbes also influence phenolic compound metabolism and bioavailability. Although preclinical studies suggest their potential in preventing and treating disorders and diseases, more clinical research is needed to confirm these benefits (Chiu et al., 2021).

#### 5.1.4 Animal and human studies

Several studies have explored the effects of probiotics and prebiotics across different models. Martinez-Porchas et al. (2023) conducted a meta-analysis revealing minimal variations in the gut microbiota structure of tilapia exposed to feed additives (probiotics, prebiotics, and biofloc) across 221 samples from multiple studies. Despite the diversity of the datasets and potential methodological biases, this comprehensive analysis identified consistent core microbiota taxa, including Proteobacteria, Fusobacteria, Actinobacteria, Firmicutes, and Bacteroidetes, suggesting a resilient microbial community capable of adapting to dietary interventions without compromising host physiological function. These findings provide foundational insight for sustainable aquaculture practices that leverage microbial modulation. Likewise, Khalil et al. (2021) found that yogurt and gum Arabic supplementation improved gut microbiota composition in rats, enhancing beneficial bacteria such as *Bifidobacterium* and *Lactobacillus*. This change in microbiota was associated with better blood glucose and lipid control, highlighting the importance of gut health in managing diabetes. Using an *in vitro* gut microbiome model, Duque et al. (2021) investigated the combined effects of the probiotic strains *Limosilactobacillus reuteri* and *Bifidobacterium longum* with the prebiotic galacto-oligosaccharides (GOS) in children with autism spectrum disorder. The treatment enhanced gastrointestinal resistance, increased *Lactobacillus* abundance, and promoted *Bifidobacterium* growth while reducing potentially harmful genera such as *Lachnoclostridium*. Importantly, prebiotic and synbiotic interventions raised short-chain fatty acid concentrations and lowered ammonium levels, indicating a favorable shift in microbial metabolism with potential implications for gut-brain axis modulation in neurodevelopmental disorders. Finally, Qureshi et al. (2024) highlighted those prebiotics and probiotics regulate gut microbiota composition in individuals with obesity by increasing beneficial bacteria while reducing harmful microorganisms. This modulation helps alleviate gut dysbiosis, which is associated with inflammation and excessive fat accumulation. By restoring microbial balance, these therapies contribute to improved metabolic health and weight management.

#### 5.1.5 Future tools like AI/ML

Machine learning enables large-scale analysis of gut microbes and prebiotic sources, allowing rational selection of substrates or synbiotic formulations based on genomic and metabolic predictions, without the need for preliminary in vitro tests. Additionally, they allow for a more precise assessment of microbiota composition and functional activity, such as bioactive metabolite production, paving the way for personalized nutrition and targeted therapeutic strategies (Sabater et al., 2021).

### 5.2 Effects on the nervous system

#### 5.2.1 Neurodevelopmental disorders

The gut microbiota communicate with the nervous system through the vagus nerve, immune signaling, and endocrine pathways. Microbial metabolites such as SCFAs, serotonin, and lipopolysaccharides influence neurotransmission, neuroinflammation, and brain function. When gut permeability increases, they can cross the blood–brain barrier and affect neural activity (Nie et al., 2024).

Microbial modulation shows promise in neurological health. Probiotic and prebiotic supplementation, individually or in combination, has shown positive effects on autism-related behaviors and molecular markers. These treatments improved social interaction, anxiety, and repetitive behaviors, while also increasing anti-inflammatory IL-10 levels. Probiotics restored the Bacteroidetes/Firmicutes ratio and reduced IL-6 levels, while the combined treatment additionally increased 5-HT levels in the prefrontal cortex (Adıgüzel et al., 2022). Similarly, *L. plantarum* OLL2712 improved memory function in older adults by reducing inflammation-associated gut bacteria and enhancing cognitive scores (Sakurai et al., 2022). After 12 weeks, participants who consumed heat-treated OLL2712 exhibited significant improvements in composite memory and visual memory scores compared to the placebo group. Additionally, the gut microbiota of the active group showed a reduced abundance of inflammation-associated genera, including *Lachnoclostridium*, *Monoglobus*, and *Oscillibacter*. These results suggest that OLL2712 may mitigate memory decline by modulating gut microbiota and reducing neuroinflammation.

#### 5.2.2 Cognitive function and memory

The impact of *L. plantarum* (probiotic), *Asparagus officinalis* extract (prebiotic), and their synbiotic combination on high-fat diet (HFD)-induced cognitive dysfunction and neurodegeneration in rats was investigated (Shahin et al., 2025). The findings indicate that these interventions effectively restored cognitive function and alleviated neurodegeneration by modulating key markers such as amyloid beta, p-tau, *α*-synuclein, and brain-derived neurotrophic factor (BDNF) in the hippocampus. Additionally, the treatments improved the disrupted lipid profile and mitigated oxidative stress, inflammation, and neurotransmitter imbalances. Notably, synbiotic treatment demonstrated superior effects by restoring gut microbiota balance, increasing beneficial *Lactobacillus* species, and reducing harmful bacteria (e.g., coliform and staphylococci), suggesting that the combined impact on gut health and brain function was more potent than the individual therapies. Flavonoids and phenolic compounds further support brain health by enhancing the production of key metabolites such as SCFAs, *γ*-aminobutyric acid (GABA), and BDNF. Certain Gram-positive bacteria, including *Lactobacillus* and *Bifidobacterium* spp., convert glutamate into GABA, a major inhibitory neurotransmitter. Additionally, phenolic compounds stimulate beneficial bacteria such as *Streptococcus*, *Escherichia,* and *Enterococcus* spp., promoting SCFA synthesis and neurotransmitter production, including serotonin via tryptophan metabolism, thereby influencing brain function through the gut-brain axis (Cano et al., 2024).

#### 5.2.3 Mood and anxiety disorders

Tae and Kim (2024) analyzed the impact of prebiotic and probiotic food consumption on anxiety in 4317 adults and found that higher intake of these foods was linked to lower anxiety levels. However, prebiotic consumption was associated with higher anxiety in both men and women, while probiotic food intake significantly reduced anxiety in men. These results suggest that prebiotics and probiotics may influence the nervous system, particularly through the gut-brain axis, with gender differences in their effects.

According to Zhao et al. (2024), the gut microbiota influence mood disorders such as major depressive disorder and bipolar disorder through the microbe-gut-brain axis, a bidirectional communication system. Gut microbes can regulate brain function, impacting mental health. Therapeutic strategies such as probiotics, prebiotics, synbiotics, and fecal microbiota transplantation may help restore microbial balance and alleviate symptoms of these disorders.

Despite promising findings, current research on probiotics, prebiotics, and synbiotics in neurodevelopmental and neuropsychiatric disorders faces several limitations. These include the lack of strain-specific evidence, variability in host response due to genetic and environmental factors, and the scarcity of long-term, large-scale human clinical trials. Furthermore, most studies rely on small sample sizes and short intervention periods, making it difficult to draw definitive conclusions regarding efficacy and safety. Addressing these gaps will require standardized protocols, extended follow-up durations, and multi-center collaborations to ensure reproducibility and generalizability of results (Marco et al., 2021).

### 5.3 Effects on the immune barrier

The gut microbiota play a crucial role in systemic immunity by modulating cytokine production, regulating immune cell activity, and strengthening the intestinal barrier (Sivan et al., 2015). Certain probiotics further enhance immune defenses by stimulating phagocytosis, activating natural killer cells, and interacting with dendritic cells (Piccioni et al., 2023). They also boost antibody production, improve vaccine responses, and promote anti-inflammatory cytokine release, potentially reducing the risk of colon cancer and colitis. Additionally, gut microbes contribute to pathogen defense through competitive exclusion, antimicrobial compound production, and nutrient metabolism, impacting overall immunity and health (Cano et al., 2024).

While the exact mechanisms by which probiotics exert their immunomodulatory effects are not yet fully understood, several potential pathways have been proposed. Probiotics are believed to influence immune function through the inhibition of Toll-Like Receptors (TLRs), which play a central role in the recognition of microbial components and the activation of inflammatory responses. By downregulating TLR expression, probiotics can reduce the activation of inflammatory pathways such as NF-κB, which is involved in the transcription of pro-inflammatory cytokines (Plaza-Diaz et al., 2019). Additionally, probiotics may modulate the activity of innate immune cells, such as Natural Killer cells, enhancing their cytotoxic potential and improving immune surveillance (Kwok et al., 2022). Probiotic supplementation has also been shown to impact oxidative stress markers, reducing oxidative damage and improving the balance between antioxidants and oxidants. By modulating factors such as nitric oxide and C-reactive protein, probiotics help mitigate the risk of inflammatory diseases, cardiovascular dysfunction, and metabolic disorders. These mechanisms highlight the potential of probiotics to regulate immune responses, although further research is needed to clarify strain-specific effects and optimal intervention strategies (Kwok et al., 2022).

While many studies report the general immunomodulatory benefits of probiotics, it is important to emphasize the significant strain specificity in their effects on immunity. Variability in host response due to genetic, environmental, and lifestyle factors further complicates the translation of findings. Moreover, there is a notable lack of long-term human clinical trials assessing safety, efficacy, and optimal dosing regimens of specific probiotic strains in diverse populations. Addressing these research gaps is crucial for advancing the clinical application of probiotics in immune-related conditions and for developing personalized probiotic therapies tailored to individual immune profiles (Kim et al., 2014; Sempach et al., 2024). Application-based studies have demonstrated the immune-enhancing potential of specific probiotic strains. For instance, *L. plantarum* supplementation in mice enhanced immune organ activity, modulated immune cell populations, and increased antimicrobial substances and immunoglobulin levels (Sivan et al., 2015). Additionally, this strain strengthens mucosal immunity while maintaining immune homeostasis, making it a promising antigen delivery carrier. It enhances antigen immunogenicity, boosts defense against harmful antigens, and has been recognized for its potential as a mucosal vaccine carrier due to its ability to modulate immune tolerance (Zare et al., 2024). Similarly, Kang et al. (2021) observed that heat-killed *Lactococcus lactis* MG5125 and various *Lactobacillus* strains suppressed nitric oxide production by up to 86.2% and reduced the expression of nitric oxide synthase and cyclooxygenase-2 induced by lipopolysaccharides. This suggests that heat-killed probiotics may offer a stable alternative to live probiotics in functional foods, while still modulating immune responses effectively.

Early-life gut microbiota composition influences allergy development. Intestinal dendritic cells regulate Treg cells, which are linked to immune tolerance. Additionally, oligosaccharide supplementation has alleviated atopic dermatitis symptoms in children, with improvements associated with changes in peripheral eosinophil levels, highlighting the immunomodulatory role of prebiotics (Kim et al., 2024). Similarly, probiotics play a crucial role in immune regulation beyond early childhood, including in physically active individuals. In athletes, probiotic supplementation has been shown to influence immune regulation in several ways. Tavakoly et al. (2021) demonstrated that probiotics modulate key immune cell populations, including reductions in T cytotoxic lymphocytes and monocytes, while multi-strain formulations increase leukocyte counts. Complementing these findings, Guo et al. (2022) reported that probiotics enhance immune defense by increasing IFN-*γ* and salivary IgA levels while reducing TNF-*α* and IL-10, particularly in short interventions. The absence of significant effects on other inflammatory markers suggests that probiotics selectively regulate immune responses, highlighting their potential role in optimizing immune function in athletes. These findings underscore the importance of selecting appropriate strains and tailoring interventions based on the target population and desired immune outcomes.

### 5.4 Metabolites and neurotransmitters

A series of microbial metabolites have been implicated in the regulation of brain function, including branched-chain amino acids, trimethylamine-N-oxide, short-chain fatty acids, tryptophan metabolites, gamma-aminobutyric acid, bile acid metabolites and choline (Meher et al., 2024). Their mode of action is principally indirect, for example by improving intestinal health, exerting anti-inflammatory effects and modulating the production of metabolites such as serotonin, leptin and insulin that affect brain function. However, they may also have a direct effect, for example through the activation of aryl hydrocarbon receptor that takes place through the production of indoles (Aoki et al., 2018; Pappolla et al., 2021). Research has mainly focused on short-chain fatty acids, tryptophan metabolites, and ghrelin as well as the impact of probiotic, prebiotic and symbiotic supplementation on their production. These compounds are important due to their protective effects against obesity, depression, anxiety, colitis, atopic dermatitis and cancer. Table 4 presents recent studies demonstrating these protective effects. These compounds also mediate gut-brain axis communication via neuronal, endocrine, and immune pathways, engaging diverse microbial, neural, hormonal, and immune mediators (Table 5). These mechanisms enable bidirectional signaling between the gut microbiota and the brain, influencing mood, cognition, behavior, and neurological health.

**Table 4:** Recent studies highlighting the protective effects of short-chain fatty acids, tryptophan metabolites and ghrelin against adverse health situations.

Studied intervention	Main outcomes	Reference
*Bt. pullicaecorum* DSM 23266 was administered to 1,2-dimethylhydrazine-induced colon tumors male BALB/cByJNarl mice.	*Bt. pullicaecorum* repressed CSE1L-induced tumorigenic potential through the production of butyric acid.	Chang et al. (2022)
A mixture of *L. plantarum* 299v (DSM9843), *S. cerevisiae* var. *boulardii* (DBVPG6763), and octacosanol were administered to obese women of reproductive age	After the intervention, c-reactive protein and IL-6 levels were significantly lower while ghrelin and HDL-cholesterol levels were significantly increased.	Okuka et al. (2024)
Adult inpatients with a current mild depressive episode received probiotic supplement consisting of eight different bacterial strains: *St. thermophilus* NCIMB 30438, *Bi. breve* NCIMB 30441, *Bi. longum* NCIMB 30435, *Bi. infantis* NCIMB 30436, *Lb. acidophilus* NCIMB 30442, *L. plantarum* NCIMB 30437, *L. paracasei* NCIMB 30439, and *Lb. delbrueckii* subsp. *bulgaricus* NCIMB 30440.	During the time of intervention, the circulating levels of ghrelin were increased and the transcription of genes functionally associated with the immune system was affected. Overall, the depressive symptoms improved during the intervention.	Sempach et al. (2024)
Prebiotic galacto-oligosaccharides were administered to Male C57BL/6 mice	Galacto-oligosaccharide supplemented diet led to the enhancement of Lachnospiraceae and *Akkermansia* populations that can metabolize tryptophan, as well as the production of tryptophan metabolites, such as indole-3-acetic acid and the methylated derivative, which reduced microglial activity, thereby reducing anxiety-like behavior.	Spencer et al. (2024)
β-glucan was fed to dextran sodium sulfate-induced colitis male C57BL/6 J mice.	*Ba. uniformis* degraded β-glucan and produced nicotinamide that promoted growth of *Lb. johnsonii*, which produced indole-3-lactic acid that activated the aryl hydrocarbon receptor, which was responsible for colitis mitigation.	Zhang et al. (2024)
Polysaccharide from vinegar-processed *Schisandra chinensis* was administered to high fat diet-induced type 2 diabetes mellitus male Kunming mice.	The polysaccharide mitigated the gut microbiota imbalance, increased the level of intestinal short-chain fatty acids, and enhanced the expression of intestinal GPR41 and GPR43 receptors, significantly enhancing the PI3K/AKT/GSK3β and AMPK/SREBP-1c/FAS signaling pathways, which resulted in a significant reduction of blood glucose and lipid levels, alleviation of pancreatic and liver injuries, repair of the intestinal barrier, and inhibition of the inflammatory response.	Guo et al. (2025)
Exopolysaccharide extracted from *Agaricus sinodeliciosus* var. Chaidam was administered on Aβ_1–42_- induced AD Sprague–Dawley rats.	The exopolysaccharide reshaped gut microbiota composition by increasing the relative abundance of Ruminococcaceae and reducing Erysipelotrichaceae, which resulted in the increase of serotonin levels in the intestinal tract of the rats, significantly alleviating cognitive deficit and neuroinflammation, potentially by enhancing microglial phagocytosis of Aβ_1–42_.	Lu et al. (2025)
*Limosilactobacillus fermentum (Li. fermentum)* 016 was administered to DSS-induced colitis of C57BL/6 J mice	*Li. fermentum* activated the the Nrf2–Keap1 signaling pathway and regulated the systemic inflammation markers, reshaped the gut microbiota by improving the microbial diversity and the abundance of beneficial bacteria, and enhanced tryptophan metabolism and the production of key metabolites with anti-inflammatory and tissue-protective effects. Overall, the colonic pathological damage and histological injury scores were reduced.	Pan et al. (2025)
Chickpea resistant starch was fed to calcipotriol-induced atopic dermatitis female Balb/c mice.	Chickpea resistant starch altered the gut microbiome and significantly increased the proportions of *Bu. virosa*, *Bi. pseudolongum* and *Fa. rodentium*. As a result, a total of 206 microbial metabolites were affected, with a notable increase of propionate and butyrate. Activation of GPR109A by acylated butyrate significantly improved the pathological characteristics.	Yan et al. (2025)
*Li. reuteri* DSM 17938 was administered to triptolide-induced liver injury male C57BL/6 J mice.	*Li. reuteri* DSM 17938 enhanced microbiota-produced propionate levels, which activated AMPK signaling that alleviated disrupted mitochondrial biogenesis and energy metabolism homeostasis, which in turn diminished ROS production and oxidative stress injury in hepatocytes.	Zhao Y, et al. (2025)

*Ba., Bacteroides*; *Bi., Bifidobacterium*; *Bt., Butyricicoccus*; *Bu., Butyricimonas*; *Fa., Faecalibaculum*; *La, Lacticaseibacillus*; *Li., Limosilactobacillus*; *Lp., Lactiplantibacillus*; *S., Saccharomyces*; *St., Streptococcus*.

**Table 5:** Key pathways of gut-brain axis communication.

Pathway	Key components/Mediators	Source/Target	Mechanism of action	Impacts on brain function/health
Neuronal	Vagus nerve, Enteric Nervous System (ENS), neurotransmitters (GABA, serotonin, dopamine, acetylcholine)	Gut microbiota → Vagus nerve → CNS	Microbial metabolites stimulate vagal afferents or modulate neurotransmitter production	Mood regulation, stress response, anxiety, depression, cognitive function
Endocrine	Hypothalamic–pituitary–adrenal (HPA) axis, cortisol, gut peptides (GLP-1, PYY, ghrelin)	Gut → Circulation → Brain	Gut microbiota modulate secretion of hormones and stress mediators	Stress reactivity, appetite regulation, emotional behavior
Immunological	Cytokines (IL-6, IL-10, TNF-α), Treg cells, gut-associated lymphoid tissue (GALT)	Gut microbiota → Immune system → Brain	Microbial signals regulate immune cell differentiation and cytokine release	Neuroinflammation, mood disorders, neurodegenerative diseases
Microbial Metabolites	Short-chain fatty acids (SCFAs: acetate, propionate, butyrate), tryptophan metabolites, secondary bile acids	Gut lumen → Circulation/ENS/BBB	Modulate blood–brain barrier integrity, epigenetics, and neuroinflammation	Neuroprotection, neurotransmission, cognitive modulation

BBB, blood–brain barrier; CNS, central nervous system; ENS, enteric nervous system; GABA, gamma-aminobutyric acid; GALT, gut-associated lymphoid tissue; GLP-1, glucagon-like peptide-1; HPA, hypothalamic–pituitary–adrenal; IL, interleukin; PYY, peptide YY; SCFAs, short-chain fatty acids; Treg cells, regulatory T cells; TNF-α, tumor necrosis factor-alpha.

### 5.5 Short-chain fatty acids

Short-chain fatty acids (SCFA) are microbial metabolites with fewer than six carbon atoms, produced in the colon. The most represented ones are acetate, propionate and butyrate; formate and lactate are also produced, but at lower quantities. In the cecum and the proximal colon there is an increased availability of fermentable substrates compared to the distal colon. These substrates include resistant starch and components of plant cell walls, which have escaped digestion in the small intestine. Thus, the concentration of SCFA tends to be higher in the proximal colon and is depleted toward the distal colon (van der Beek et al., 2017). SCFA concentrations are estimated at 70 and 140 mM in the proximal colon and 20–70 mM in the distal colon (Wong et al., 2006). Their ratio along the colon is similar, namely 3:1:1 (acetate:propionate:butyrate), which reflects their efficient and concentration-dependent absorption (Topping and Clifton, 2001). The factors that affect qualitatively and quantitatively the production of SCFA are the ones that affect the composition of the microecosystem and the metabolic activity of the producer microorganisms, such as diet (Flint et al., 2015; Garcia-Mantrana et al., 2018; Selma-Royo et al., 2019; Fusco et al., 2023; Yi et al., 2025), gut transit time (Wong et al., 2006) pH value (Wong et al., 2006; Yi et al., 2025) and bile salt concentration (Flint et al., 2015).

The pathways for acetate production, namely acetogenesis and the Wood-Ljungdahl pathway, seem to be widely distributed among the phyla that comprise the human gut microbiome. On the contrary, production of butyrate and propionate is substrate specific, and the respective pathways seem to be restricted to a few species. Butyrate is mainly produced through the CoA-transferase pathway and only a few species use the butyrate kinase pathway (Flint et al., 2015). Butyrate production via the CoA-transferase pathway is mainly driven by *Eubacterium hallii*, *Eubacterium rectale*, *Faecalibacterium prausnitzii*, *Roseburia faecis*, and other *Lachnospiraceae* species (Louis et al., 2010; Reichardt et al., 2014). On the other hand, the occurrence of the butyrate kinase pathway has only been reported in *Coprococcus eutactus* and *Coprococcus comes* (Reichardt et al., 2014). Interestingly, the presence and metabolic activity of *Ruminococcus bromii* is particularly important when resistant starch is available (Ze et al., 2012). On the other hand, propionate can be produced by three pathways, the succinate pathway used by Bacteroidetes and some Firmicutes, the propanediol pathway that operates only when fucose and rhamnose serve as carbon sources and is used by some members of the *Lachnospiraceae* family, and the acrylate pathway that is restricted to only a few members of the Firmicutes and is used to convert lactate to propionate (Reichardt et al., 2014; Flint et al., 2015).

As far as formate and lactate are concerned, the first is mainly produced by bifidobacteria and *Eubacterium hallii* (Schwab et al., 2017) while the second mainly by bifidobacteria, lactobacilli, streptococci and staphylococci (Jost et al., 2012; Pham et al., 2016). The lactate is then catabolized by bacteria such as *Eubacterium hallii* and *Anaerostipes caccae* toward the production of propionate and butyrate (Reichardt et al., 2014).

SCFA are considered as possible mediators of the communication between gut microbiota and the brain (Dalile et al., 2019). SCFA produced in the colon are rapidly absorbed by the colonocytes and used for energy production (Schonfeld and Wojtczak, 2016). The ones that are not catabolized in the colonocytes are transported to the liver, where they are used for energy production by the hepatocytes, with the exception of propionate that can also be used for gluconeogenesis and acetate that can also be used to produce fatty acids and cholesterol (Boets et al., 2017). As a result, only a small percentage of the SCFA produced in the colon can reach peripheral organs through systemic circulation, which has been calculated at 2, 9 and 36% for butyrate, propionate and acetate, respectively (Boets et al., 2015). SCFA modulate brain function through immune, endocrine and vagal pathways (Dalile et al., 2019). The interactions of SCFA with a variety of immune cells may modulate brain activity. More specifically, SCFA directly affect neutrophils by regulating the production of inflammatory cytokines and by acting as neutrophil chemoattractants (Rodrigues et al., 2016), inhibit the maturation of monocytes, macrophages and dendritic cells (Correa-Oliveira et al., 2016; Chang et al., 2014) and affect the differentiation and proliferation of T cells (Kim et al., 2014). Although a variety of mechanisms have been proposed, the inhibition of histone deacetylases appears to play a key role. The endocrine pathway is activated by the secretion of gastrointestinal as well as other metabolic hormones, which is affected by colonic SCFA. The production of SCFA in the colon activates orphan G protein-coupled receptors which results in the production of peptide tyrosine tyrosine (PYY) and glucagon-like peptide 1 (GLP1) by enteroendocrine L cells (Tolhurst et al., 2012; Larraufie et al., 2018). On the other hand, there are indications that the production of hormones such as leptin, ghrelin and insulin is modulated by colonic SCFA (Xiong et al., 2004; Robertson et al., 2005; Rahat-Rozenbloom et al., 2017); however, the underlying mechanism is yet to be fully elucidated. The mechanisms by which these hormones affect brain function have been extensively assessed, and especially in the case of PYY and GLP1, have already been proposed (Koda et al., 2005; Katsurada and Yada, 2016). In the case οf ghrelin, SCFA have been reported to interfere with ghrelinergic signaling, most likely by antagonistic binding to its receptor GHSR-1a (Torres-Fuentes et al., 2019). Indications that SCFA stimulate the vagal afferents have been repeatedly reported (Bercik et al., 2011; Bravo et al., 2011; Goswami et al., 2018). This activation may be mediated by the free fatty acid receptor 3 (FFAR3; Bonaz et al., 2018) that is expressed in nodose ganglion neurons (Nohr et al., 2015).

The aforementioned interactions of SCFA are considered as the main mechanisms through which they contribute to the reduction of symptom severity or treatment of gastrointestinal, metabolic, cardiovascular, neurological and other disorders that have been associated with the gut-brain axis (Xiong et al., 2022; Zhang et al., 2023; Facchin et al., 2024).

### 5.6 Tryptophan and metabolites

Tryptophan is an essential amino acid; therefore, humans rely on dietary intake. Dietary tryptophan is mainly used for protein synthesis. Free tryptophan, i.e., the tryptophan that is not used for protein synthesis is mainly catabolized through the kynurenine pathway to produce a wide range of biologically active metabolites, collectively termed kynurenines. Over 95% of free tryptophan catabolism occurs via this pathway (Polyzos and Ketelhuth, 2015). The kynurenines have wide physiological and often opposing roles that are essential in immune responses, inflammation, oxidative stress and neurodegeneration, affecting, thus, brain function (Tanaka et al., 2024). Tryptophan may also be used for the biosynthesis of serotonin and melatonin, the modulation of the brain function by both has been well documented (Carhart-Harris and Nutt, 2017; Lee et al., 2019). Finally, the gut microbiota may use tryptophan for the production of indoles and their derivatives. Colonic microbiota may shift from saccharolytic to proteolytic metabolism-depending on protein intake, carbohydrate availability, transit time, and pH-leading to protein degradation and tryptophan catabolism. This catabolic shift has been reported as more intense toward the distal colon (Smith and Macfarlane, 1996; Geypens et al., 1997; Zelante et al., 2013; Roager et al., 2016; Vieira-Silva et al., 2016). The capacity of several Gram-positive and -negative species to catabolize tryptophan and produce indoles and their derivatives has been reported; most of them belong to the genera *Anaerostipes*, *Bacteroides*, *Bifidobacterium*, *Butyrivibrio*, *Clostridium*, *Desulfovibrio*, *Enteroroccus*, *Escherichia*, *Eubacterium*, *Faecalibacterium*, *Fusobacterium*, *Haemophilus*, *Lactobacillus* sensu lato, *Megamonas*, *Parabacteroides*, *Peptostreptococcus* and *Ruminococcus* (Roager and Licht, 2018).

The gut microbiota may affect directly or indirectly tryptophan metabolism by the host. The direct effect may result from the reduction of tryptophan availability for the host, which may lead to decreased serotonin and 5-hydroxyindoleacetic acid production, which, in turn, may lead to depressive-like behavior (Lukic et al., 2019). Interestingly, a causal link has been suggested, as depressive phenotypes can be transferred via gut microbiota transplantation (Kelly et al., 2016). Serotonin production may also be modulated, either toward stimulation that has been reported to occur by spore-forming bacteria including *Clostridium ramosum* (Yano et al., 2015; Mandic et al., 2019), or toward disruption (Golubeva et al., 2017). Similarly, modulation of the kynurenine pathway may also take place, as in the case of *Lactobacillus johnsonii* N6.2, which reduced the production of indoleamine-2,3-deoxygenase that catalyzes the oxidation of L-tryptophan to N-formylkynurenine, the first step of the kynurenine pathway (Valladares et al., 2013). The indirect effect has been reported to occur either through butyrate production, which has been reported to suppress kynurenine production (Martin-Gallausiaux et al., 2018), or through the maintenance of gut integrity that may prevent gastrointestinal disorders, such as inflammatory bowel disease and irritable bowel syndrome, which have been associated with disruption of the serotonergic signaling pathways (Gracie et al., 2019). Maintenance of gut integrity can be achieved through a number of mechanisms including the promotion of cytokine release, such as IL-6, IL-17 and IL-22, by indole derivatives through the activation of the aryl hydrocarbon receptor (Zelante et al., 2013; Schiering et al., 2017; Busbee et al., 2020; Zhang et al., 2024).

These direct and indirect effects of gut microbiota on tryptophan metabolism by the host regulate intestinal and systemic homeostasis in both health and disease (Zhao P, et al., 2025). More specifically, the development of many diseases including digestion, respiratory, blood, neoplastic and non-neoplastic ones has been associated with disruption of tryptophan metabolism. Therefore, the therapeutic potential of restoration of tryptophan metabolism has been indicated (Platten et al., 2019; Chen et al., 2024).

### 5.7 Ghrelin

Ghrelin is a 28 amino acid hormone primarily produced in the stomach. The acylated form of ghrelin binds with high affinity to the growth hormone secretagogue receptor (GHSR), and more specifically GHSR-1a. This receptor is ubiquitously expressed in central and peripheral nervous system and has been implicated in the regulation of an extended array of functions related to feeding behavior, metabolism and energy storage. Therefore, it is considered as a key molecule that communicates nutrition-related information along the gut-brain axis (Leeuwendaal et al., 2021).

Studies report both positive and negative correlations between gut microbiota and ghrelin levels (Parnell and Reimer, 2012; Hooda et al., 2013; Gomez-Arango et al., 2016; Kang et al., 2016; Liu et al., 2017; Massot-Cladera et al., 2017; Yanagi et al., 2017; Yang et al., 2019; Bo et al., 2019) suggesting a regulatory relationship (Mahana et al., 2016; Ikenoya et al., 2018). More specifically, ghrelin levels seem to be affected by the lipopolysaccharides of Gram-negative bacteria, as well as by metabolites such as formylated peptides, amino acids, hydrogen sulfide and SCFA. The first has been adequately exhibited in the case of *Helicobacter pylori*, whose lipopolysaccharide seems to activate an inflammatory response through TRL-4 stimulation and ghrelin-mediated GHSR-1a activation (Slomiany and Slomiany, 2017). Similarly, formylated peptides may also have an indirect effect on ghrelin levels as they activate the epithelial GPCR formyl peptide receptor 1 (FPR1) stimulating ROS generation by epithelial cells, which in turns increases plasma ghrelin concentration (Suzuki et al., 2011; Alam et al., 2014). Microbial proteolysis of dietary proteins produces amino acids that affect plasma ghrelin levels in a residue-specific manner. More specifically, L-glutamine, L-glutamic acid, L-lysine, L-threonine and L-valine increase ghrelin plasma levels while L-cysteine, L-leucine and L-tryptophan reduces them (McGavigan et al., 2015; Steinert et al., 2017; Elsabagh et al., 2018; Yin et al., 2018a, 2018b). L-cysteine holds an additional role as its degradation is the major pathway for hydrogen sulfide production. The latter has been reported to negatively affect the ghrelin secretion (Slade et al., 2018). A negative correlation has also been reported between SCFAs and ghrelin levels (Rahat-Rozenbloom et al., 2017). Two mechanisms have been proposed, a direct that includes antagonism for the GHSR-1a receptor and an indirect that includes FFAR2-mediated regulation (Torres-Fuentes et al., 2019).

## 6 Current challenges and future directions

Although evidence on probiotics, prebiotics, and synbiotics in modulating the gut-brain axis (GBA) is growing, key challenges still hinder the translation of preclinical findings into clinical practice. First, heterogeneity in dosage, duration, and strain selection complicates interpretation and undermines reproducibility. Second, many studies are conducted in small, homogeneous populations, reducing the generalizability of findings across diverse age groups, lifestyles, and clinical conditions. Third, although animal studies provide valuable mechanistic insights, their predictive value for human physiology and neurocognitive outcomes remains limited. Moreover, findings are inconsistent: some studies show clear benefits while others report null or adverse outcomes, fueling ongoing debate.

Another critical limitation is the lack of standardized biomarkers and validated clinical endpoints for assessing GBA-related benefits. Current measures mainly depend on subjective self-reports or indirect proxies, which fail to fully capture the multidimensional nature of gut-brain interactions. In addition, the long-term safety and efficacy of chronic probiotic or synbiotic supplementation are still unclear, as most trials are of relatively short duration.

Future research should prioritize large-scale, well-controlled, and multi-center trials with clearly defined outcomes to establish clinical relevance. Integrating omics technologies, such as metabolomics, metagenomics, and transcriptomics, may provide a systems-level understanding of host-microbiome interactions. Furthermore, there is a need to explore personalized approaches, as individual differences in microbiome composition, genetics, and lifestyle factors likely influence responsiveness to interventions. Addressing these challenges is essential to translate promising findings into evidence-based strategies for brain and systemic health.

## 7 Conclusion

Studies confirm that the gut microbiota play an essential function in regulating the two-way communication between the gastrointestinal tract and the central nervous system, commonly referred to as the gut-brain axis. Probiotics, prebiotics, and synbiotics modulate the gut-brain axis by influencing microbial composition, metabolic activity, immune responses, and neurochemical pathways. Studies suggest that their inclusion in the diet may alleviate clinical manifestations of various neurological and psychiatric disorders, enhance cognitive function and improve systemic immune function. However, key issues must be resolved before clinical implementation. Upcoming research must give priority to clarifying how hosts and microbiota interact, variability between microbial strains and substrates, the influence of individual genetic and environmental factors, and standardized clinical trials should be performed. Large-scale randomized trials integrating multi-omics approaches are needed to clarify the functional effects of these interventions. In addition, personalized approaches that take into account individual variability in microbiota composition, genetics, diet and lifestyle are important to maximize therapeutic efficacy and minimize adverse outcomes. It is also important to focus on optimizing microbial formulations and defining precise therapeutic windows for different disease states. Additionally, emerging technologies such as machine learning and systems biology will open up different opportunities to predict host responses and design tailored microbial therapies. Moreover, the development of next-generation probiotics and designer synbiotics targeting specific pathways involved in neuroinflammation, neurotransmitter synthesis and immune modulation represents a promising direction for future research. In conclusion, regulating the gut-brain axis is a promising strategy for managing neurological, psychiatric, and systemic diseases. Interdisciplinary research and technological advances are crucial for translating findings into clinical practice.
